# Nanosurface‐Reconstructed Fuel Electrode by Selective Etching for Highly Efficient and Stable Solid Oxide Cells

**DOI:** 10.1002/advs.202409272

**Published:** 2024-12-03

**Authors:** Yueyue Sun, Jun Zhou, Jiaming Yang, Dragos Neagu, Zhengrong Liu, Chaofan Yin, Zixuan Xue, Zilin Zhou, Jiajia Cui, Kai Wu

**Affiliations:** ^1^ Center of Nanomaterials for Renewable Energy State Key Laboratory of Electrical Insulation and Power Equipment Xi'an Jiaotong University Xi'an 710049 P. R. China; ^2^ Xi'an Thermal Power Research Institute Co., Ltd Xi'an 710054 P. R. China; ^3^ Department of Chemical and Process Engineering University of Strathclyde Glasgow G1 1XL UK; ^4^ School of Materials Science and Engineering Xi'an University of Technology Xi'an 710048 P. R. China

**Keywords:** acid etching, CO_2_ electrolysis, exsolution, fuel electrode, solid oxide cells

## Abstract

Solid oxide cells (SOCs) are promising energy‐conversion devices due to their high efficiency under flexible operational modes. Yet, the sluggish kinetics of fuel electrodes remain a major obstacle to their practical applications. Since the electrochemically active region only extends a few micrometers, manipulating surface architecture is vital to endow highly efficient and stable fuel electrodes for SOCs. Herein, a simple selective etching method of nanosurface reconstruction is reported to achieve catalytically optimized hierarchical morphology for boosting the SOCs under different operational modes simultaneously. The selective etching can create many corrosion pits and exposure of more B‐site active atoms in Sr_2_Co_0.4_Fe_1.2_Mo_0.4_O_6‐δ_ fuel electrode, as well as promote the exsolution of CoFe alloy nanoparticles. An outstanding electrochemical performance of the fabricated cell with the power density increased by 1.47 times to 1.31 W cm^−2^ at fuel cell mode is demonstrated, while the current density reaches 1.85 A cm^−2^ under 1.6 V at CO_2_ electrolysis mode (800 °C). This novel selective etching method in perovskite oxides provides an appealing strategy to fabricate hierarchical electrocatalysts for highly efficient and stable SOCs with broad implications for clean energy systems and CO_2_ utilization.

## Graphical abstract

Manipulating surface architecture is vital for the electrochemical materials. Herein, acid etching reconstructs the perovskite fuel electrode surface, including corrosion pits, exposure of active B‐site cations, and surface A‐site deficiency. This novel and active hierarchical surface with exsolved CoFe nanoparticles presents enhanced solid oxide fuel cells and CO_2_ electrolysis performance, providing a simple and efficient strategy for designing highly active perovskite catalysts.

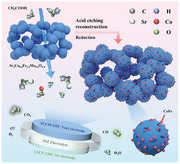

## 1 Introduction

Solid oxide cells (SOCs) are electrochemical energy conversion devices that have demonstrated very high efficiency in both converting fuels to electricity (fuel cell mode, FC mode) and storing electricity as chemical fuels (electrolysis cell mode, EC mode).^[^ ^1^ ^]^ The fuel electrode is crucial for both the hydrogen oxidation reaction (HOR) under FC mode and carbon dioxide reduction reaction (CO_2_RR) under EC mode. Sluggish reactivity in either process can significantly impact SOCs performance. Perovskites and Ni/cubic fluorite structures oxides are leading materials for fuel electrodes.^[^ ^2^ ^]^ However, when combined with Ni as a composite electrode, the poor coking resistance of Ni obstructs surface active sites and hampers electron transfer.^[^ ^3^ ^]^ Perovskite oxides offer various advantages such as compositional flexibility, activity in electrochemical/photoelectric processes and stability, making them a superior choice for bifunctional fuel electrodes in SOCs.^[^ ^4^ ^]^

The mixed ionic‐electronic conducting perovskite oxides exhibit excellent oxygen ion conduction and electron conduction, however, it lacks sufficient catalytic properties. Taking into account the effect of surface features on the reactivity and stability of the material, surface design or modification is required, including deposition, infiltration, and exsolution. Infiltration and electrochemical deposition enhance the fuel electrode catalysis by adding external surface catalyzer, offering advantages of limitless modified area and high growth rate.^[^ ^5^ ^]^ However, the weak attachment of the nano‐decorations results in instability because they tend to agglomerate at operating temperature due to large surface energy associated with the nanosized particles.^[^ ^6^ ^]^ Exsolution is a more stable and efficient strategy than infiltration due to its inherent socketed nature.^[^ ^6a^ ^]^ The exsolution process of various cations from bulk perovskites involves stages such as bulk diffusion, surface nucleation, and growth.^[^ ^7^ ^]^ Typical strategies for promoting exsolution include A‐site deficiency,^[^ ^8^ ^]^ B‐site cation trigger dopants,^[^ ^9^ ^]^ lattice strain control,^[^ ^10^ ^]^ etc. Nevertheless, some obstacles still exist and resist the nanoparticles (NPs) exsolution process, like surface segregation oxides, instability from excessive A‐site deficiency, and limits on B‐site exsolution, which further result in insufficient performance of fuel electrode in SOCs.^[^ ^11^ ^]^

Acid/alkali etching has multiple surface modification effects, including selectively exposing active B‐site cations, reconstructing A‐site deficient surface and creating corrosion pits. Peng et al. published that dilute HNO_3_ selectively etched Sr from La_0.5_Sr_0.5_CoO_3_, increasing the Co^3+^/Co^2+^ redox couples and catalytic performance.^[^ ^12^ ^]^ Ma et al. also used nitric acid to etch LaFeO_3_ catalysis and fabricated some corrosion pits on surface.^[^ ^13^ ^]^ This surface modification method was also used to reconstruct the SOCs electrodes. Chi et al. reported that (Co*_x_* Fe_1‐_ *_x_*)_3_O_4_ NPs were exsoluted on the nitric acid etched Pr_0.2_Sr_0.8_Co_0.2_Fe_0.8_O_3‐δ_ symmetrical electrode. CoFe alloy and CoO NPs embedded on the anode after reduction, demonstrating good SOCs performance.^[^ ^14^ ^]^ To further determine the selective surface reconstruction of acid etching, Li et al. used X‐ray absorption spectroscopy (XAS) to analyze the suitable unsaturated coordination structure and Co─O bond covalency after acid etching.^[^ ^15^ ^]^ Acid etching primarily targets A‐site cations and reconstructs an A‐deficient surface because of the longer and weaker A─O bond in perovskite, perfectly matching the preference conditions of exsolution, which has not been investigated so far.

SrFe*_x_* Mo_1‐_ *_x_* O_3‐δ_ based perovskite fuel electrodes exhibit excellent electrochemical catalytic activity and flexible exsolution in content with various lattice structures, including single/double perovskite (SP/DP) and Ruddlesden‐Popper (RP) phases. Exsolutions of NPs, such as Ni, Fe, Ru, CoFe, NiFe, and FeCoNiCu quaternary alloy, have been widely studied, showing high performance in both FC and EC modes.^[^ ^16^ ^]^ Hu et al.^[^ ^16f^ ^]^ designed a Ru@Ru‐Sr_2_Fe_1.5_Mo_0.5_O_6‐δ_ (SFM)/Ru‐Gd_0.1_Ce_0.9_O_2‐δ_ (GDC) anode. These metal‐oxide heterointerfaces have higher intrinsic activities for H_2_ power generation and CH_4_ conversion. While noble metal costs much more than base metal which can also perform great. He et al.^[^ ^16d^ ^]^ chose Cu as a dopant for SFM for both HOR and CO_2_RR. After reduction, Sr_2_Fe_1.5_Mo_0.3_Cu_0.2_O_6‐δ_ electrode is elegantly reconstructed into three phases of DP, RP, and CuFe metals, showing a 1.51 W cm^−2^ power density in FC mode and 1.94 A cm^−2^ at 1.4 V in EC mode. These studies demonstrate that SrFe_x_Mo_1‐x_O_3‐δ_ based perovskite oxides are potential materials as fuel electrode in SOCs.

Here we report an efficient and simple acid etching method that reconstructs a hierarchical catalytic surface on Sr_2_Co_0.4_Fe_1.2_Mo_0.4_O_6‐δ_ (SCFM) perovskite fuel electrode, significantly improving the performance under both FC and EC modes. This hierarchical structure involves exposed active B‐site cations, more catalytic site by corrosion pits, and boosted exsolution of CoFe alloy NPs by surface A‐site deficiency. Detailed element and coordination environment information indicates that surface segregation is resisted and oxygen vacancies are enhanced. This nanosurface reconstruction driven by acid etching improves the gas ad/desorption and electron transfer, enhancing the SOCs performance under both modes. This simple and efficient surface reconstruction method shows inspiring prospect in promoting the fuel electrode performance.

## 2 Results and Discussion

### 2.1 Structure and Morphology Characterization

**Figure 1:**
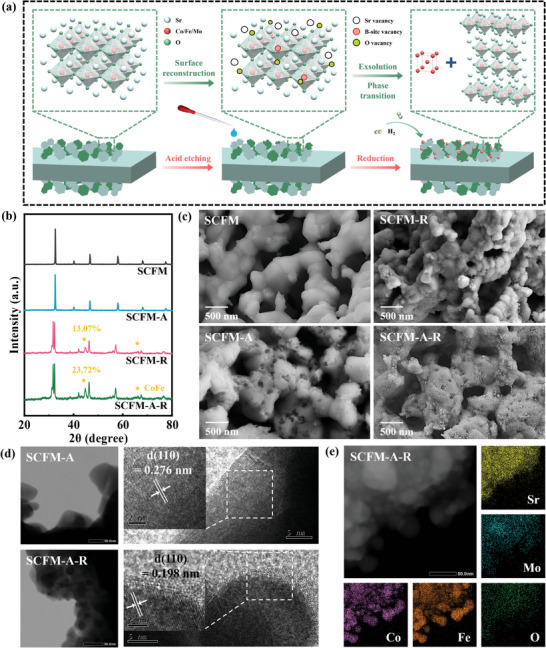
a) Schematic of the surface reconstruction of acid etching and phase transition after reduction. b) XRD patterns of the prepared SCFM, SCFM‐A, SCFM‐R, and SCFM‐A‐R. c) SEM images of different samples. d) TEM and HRTEM images for the SCFM‐A and SCFM‐A‐R with CoFe NPs. e) EDS mapping images of SCFM‐A‐R.

The fuel electrode samples are synthesized using the sol‐gel method. The acid‐etched sample is denoted as SCFM‐A, while the reduced samples are labeled as SCFM‐R and SCFM‐A‐R. **Figure** **1**a illustrates the material treatment process and the corresponding changes in lattice structure. Acid etching not only alters the surface morphology, but also reconstructs an A‐deficient nanosurface and promotes the exsolution of CoFe NPs. Figure 1b presents the X‐ray diffraction (XRD) results for the four samples. Both SCFM and SCFM‐A exhibit a *Pm*‐3*m* double perovskite structure. Post acid‐etching, SCFM‐A retains the original structure without impurities, while the XRD peak position shows a slight move to lower degree (Figure S1, Supporting Information), indicating a lattice expansion. After the reduction in wet H_2_ at 800 °C for 2 h, a phase transition occurs from the *Pm*‐3*m* perovskite phase to a combination of *I*4/*mmm* RP perovskite and exsolved *Pm*‐3*m* CoFe alloy. The phase compositions and unit cell parameters are confirmed by XRD Rietveld refinement results in Figure S2 and Table S1 (Supporting Information). The calculated content of CoFe alloy in SCFM‐A‐R is 23.7 wt%, significantly higher than the 13.1 wt% in SCFM‐R. In situ Raman spectroscopy, conducted under 5% H_2_/N_2_ from 50 °C to 600 °C, reveals only a weak peak at ≈810 cm^−1^, indicating that SCFM has a non‐ideal cubic structure (Figure S3, Supporting Information).^[^ ^17^ ^]^ The phase transition and exsolution can be described as the following equation:

(1) $$\begin{array}{ccc} 3{\mathrm{Sr}}_{2}{\mathrm{Co}}_{0.4}{\mathrm{Fe}}_{1.2}{\mathrm{Mo}}_{0.4}O_{6-\delta } & + & 4H_{2}\to 2{\mathrm{Sr}}_{3}{\mathrm{Co}}_{0.1}{\mathrm{Fe}}_{1.3}{\mathrm{Mo}}_{0.6}O_{7-\delta }\\ & & +\mathrm{Co}-\mathrm{Fe}+4H_{2}O \end{array}$$

The impact of acid etching is more clearly observed in the scanning electron microscope (SEM) images in Figure 1c and Figure S4 (Supporting Information). The SCFM powder exhibits a smooth surface, while SCFM‐A shows noticeable etching pits ranging from 20 to 100 nm, particularly near grain boundaries. After reduction, CoFe alloy NPs are uniformly exsolved and embedded in both SCFM‐R and SCFM‐A‐R, with faint traces of acid etching still visible in SCFM‐A‐R. Detailed morphology and elemental distribution are obtained through transmission electron microscope with energy dispersive spectrometer (TEM‐EDS), as shown in Figure 1d,e, and Figure S5 (Supporting Information). Before reduction, the lattice spacings of SCFM and SCFM‐A are 0.274 and 0.276 nm for the (110) plane.^[^ ^18^ ^]^ The exsolved CoFe NPs have lattice spacings of 0.201 and 0.198 nm, which closely match the d(110) of the *Pm*‐3*m* CoFe alloy phase.^[^ ^19^ ^]^ EDS images indicate a distinct exsolution of CoFe alloy NPs with a narrow size of 20 nm.

### 2.2 Selective Surface Reconstruction by Acid Etching

Inductively coupled plasma atomic emission spectroscopy (ICP‐OES) analysis was conducted to illustrate the effect of acid etching on the sample elements. Table S2 (Supporting Information) lists the weight percentages of the elements in the washed powders. The most easily etched element is Sr, followed by Co, whereas Fe and Mo are hard to be etched by acid. Additionally, the surface Sr species (such as SrO and SrCO_3_) could be etched or washed away, thus the elemental compositions of the washing solutions were also analyzed, as shown in Table S3 (Supporting Information).^[^ ^20^ ^]^ Comparing the two solutions, we found that Sr is still the dominant etched element. These results indicate that glacial acetic acid primarily etches the surface lattice Sr and Co. Assuming Fe remains unchanged due to its minimal etching, the chemical formulas of SCFM and SCFM‐A are calculated as Sr_2.01_Co_0.41_1Fe_1.20_Mo_0.43_O_6‐δ_ and Sr_1.95_Co_0.39_Fe_1.20_Mo_0.42_O_6‐δ_ respectively, based on the powders results in Table S2 (Supporting Information). The A/B ratio decreases from 0.99 to 0.97 after acid etching, indicating a slight A‐site deficiency. This deficiency is relatively small, however, the acid etching mainly reconstructs the perovskite surface, leading to highly A‐deficient surface that could enhance the exsolution, especially during the nucleation stage under a reduction atmosphere.

X‐ray photoelectron spectra (XPS) analysis provided additional insights into the surface element contents, as listed in Table S4 (Supporting Information). The A/B ratios of both SCFM and SCFM‐A are above 1 due to the presence of surface Sr species. Meanwhile, the A/B ratio drops from 1.85 to 1.25 after acid etching due to the most etched Sr. The ICP‐OES and XPS results confirm that acid etching leads to an A‐deficient fuel electrode surface. Although A‐deficiency induced by acid etching has been studied in other catalytic perovskite materials, such as LaCoO_3_, La_0.5_Sr_0.5_CoO_3_, LaFeO_3_, etc., but by the HNO_3_ solution and without exsolution phenomenon.^[^ ^12^, ^13^, ^21^ ^]^

**Figure 2:**
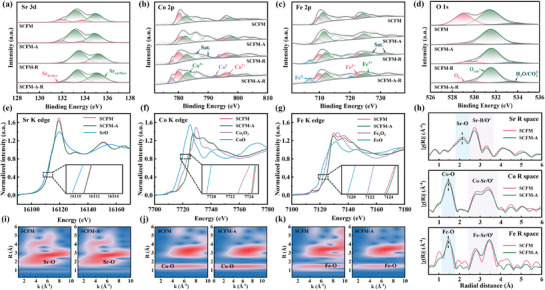
XPS spectra of the four samples taken at a) Sr 3d, b) Co 2p, c) Fe 2p, and d) O 1s. XANES spectra of the e) Sr K‐edge, f) Co K‐edge, and g) Fe K‐edge. h) R space Fourier transforms of the *k* ^3^‐weighted Sr, Co, Fe K‐edge EXAFS. i–k) Corresponding wavelet transform of the EXAFS spectra.

XPS spectra of Sr 3d, Co 2p, Fe 2p, O 1s, and Mo 3d peaks at room temperature are shown in **Figure** **2**a–d and Figure S6 (Supporting Information), with the respective proportions listed in Table S5 (Supporting Information). The peak of binding energy of surface Sr species are located at ≈132.0 eV and lattice Sr at ≈133.2 eV.^[^ ^16^, ^22^ ^]^ The proportion of lattice Sr decreases from 15% to 11% after acid etching, with both surface and lattice Sr peak intensities decreasing. Combined with the ICP‐OES and the XPS elemental results, these evidences demonstrate that acid primarily etches the lattice Sr and dissolves surface Sr species. The peaks of binding energy located at ≈529.2, ≈531.5, and ≈534.4 eV, respectively represent lattice oxygen, absorbed oxygen, and surface adsorbed molecules.^[^ ^23^ ^]^ The absorbed oxygen increasing from 59% to 84% reveals the formation of oxygen vacancies due to charge compensation during acid etching cation removal. Perovskites are usually covered by the inert A─O oxide layer and end on (001).^[^ ^11^, ^24^ ^]^ Acid initially targets the surface Sr species and bulk Sr─O in the perovskite, followed by Co─O and others, creating shallow pits on the surface, especially at grain boundaries.^[^ ^13^, ^21^ ^]^

Fe and Co XPS results indicate mixed oxidation states: Fe^3+^(≈712.1 eV, ≈724.8 eV), Fe^2+^(≈710.1 eV, ≈722.7 eV), Fe^0^(≈706.9 eV, ≈720.4 eV), Co^3+^(≈783.1 eV, ≈798.5 eV), Co^2+^(≈780.2 eV, ≈795.9 eV), and Co^0^(≈777.8 eV, ≈792.5 eV).^[^ ^19^, ^25^ ^]^ From the proportion of different valence states in Table S5 (Supporting Information), the valence states of Fe shows a slight increase, while Co shows a slight decrease.^[^ ^26^ ^]^ Surface A‐site deficiency and partially etched Co may lead to increased Fe valence states and more oxygen vacancies. A‐site deficiency and oxygen vacancies are typical strategies to boost NPs exsolution. Consequently, SCFM‐A‐R shows more Fe^0^ and Co^0^ than SCFM‐R, with Fe^0^ increasing from 15% to 23% and Co^0^ from 10% to 14%. These results are consistent with the previous XRD results.

To further analyze the detailed effect of acid etching, XAS was conducted to gain more information about the changes in the metal oxidation states and coordination environments. XAS includes two main regions: the X‐ray absorption near‐edge structure (XANES) and extended X‐ray absorption fine structure (EXAFS). The XANES region (≈40 eV around the absorption edge) provides information about oxidation state and chemical bonding environments, while the EXAFS region (extending up to 1000 eV beyond the absorption edge) offers geometric information about the absorbing atom by modeling, including distances and coordination numbers.^[^ ^27^ ^]^ Figure 2e–g illustrates the K‐edge XANES spectrums for Sr, Co, and Fe, with embedded enlarged images. The highest main resonance peak corresponds to dipolar transitions whose shape is due to the local geometrical structure.^[^ ^28^ ^]^ From all K‐edge XANES spectrums, SCFM and SCFM‐A present similar features in terms of both intensity and position, which indicates that acid etching does not alter the lattice framework.^[^ ^29^ ^]^ However, the energy shifts in the embedded images reveal a slight decrease in Co valence and an increase in Fe valence.^[^ ^30^ ^]^ Compared with standard oxides, the average valence state of Co is between 2+ and 3+, while Fe is higher than 3+.

The R space Fourier transform (FT) *k* ^3^‐weighted K‐edge EXAFS data (without phase correction) is shown in Figure 2h. Concentration mainly on the analysis of the first peak is settled in this work, because it is isolated wsell and corresponds to the first coordination shell of the center atom. For all three elements, the first coordination shell is with oxygen. As the coordination number of Sr─O, Co─O, and Fe─O shells declines, the intensity of these first peaks decreases, indicating the presence of more oxygen vacancies.^[^ ^25^, ^27^ ^]^ The amplitude of the second shell of Fe and Co is nearly half of that of the first shell, suggesting deviations from a linear B─O─B configuration due to rotations of BO_6_ octahedra or off‐center displacement of metal ions, consistent with Raman results indicating lattice distortion.^[^ ^31^ ^]^ Further fitting curves, results, and parameters are shown in Figure S7 and Table S6 (Supporting Information). The coordination numbers of oxygen in FeO_6_ and CoO_6_ octahedra complex decrease from 5.03 and 5.15 to 4.84 and 4.99 after acid etching, suggesting the increased concentration of oxygen vacancies. The Sr─O coordination number also decreases from 11.98 to 11.16. These changes could be intuitively seen from the wavelet transform graphs in Figure 2i–k, especially the Fe─O. Meanwhile, the bond distances show no obvious changes, reflecting a robust lattice framework and minor valence state changes. Acid etching reconstructs the surface and induces the loss of coordinated oxygen around A‐site and B‐site cations, which serves as the driving force for in‐situ exsolution of NPs.^[^ ^32^ ^]^

The acid selectively etches SCFM perovskite surface through a multi‐step mechanism. Firstly, preferential removal of Sr species from the surface creates an A‐site deficient layer and exposure of catalytically active B‐site cations. Etching also creates nanoscale pits, dramatically increasing the surface roughness and active surface area. The A‐site deficiency leads to the formation of additional oxygen vacancies due to charge compensation, which promotes the exsolution of CoFe NPs during reduction.

### 2.3 Fuel Cell Mode Performance

**Figure 3:**
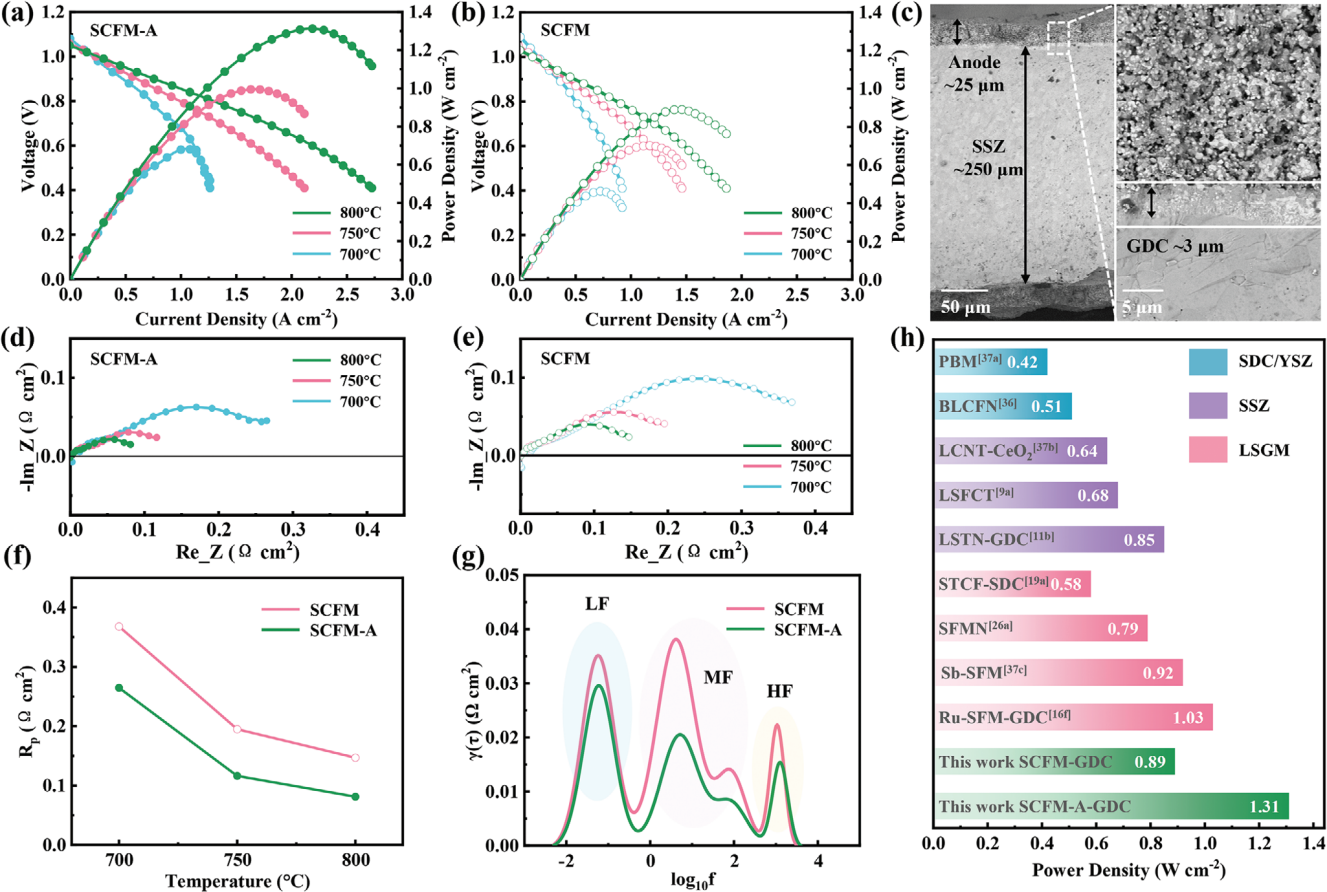
Single cell FC mode performance: a) *I*–*V*–*P* curves with SCFM‐A as fuel electrode. b) *I*–*V*–*P* curves with SCFM as fuel electrode. c) SEM images of the single cell. d) Nyquist plots for EIS with SCFM‐A as fuel electrode. e) Nyquist plots for EIS with SCFM as fuel electrode. f) Comparison of the polarization resistances. g) DRT results. h) Comparison in power density obtained at 800 °C.

The single cell SCFM‐GDC|GDC|SSZ|GDC|LSCF‐GDC was initially tested under fuel cell mode. The composite electrode SCFM‐GDC was calcined for 20 h at 1000 °C, which shows good chemical compatibility demonstrated by the XRD results in Figure S8 (Supporting Information). In **Figure** **3**c, the SEM image reveals dense SSZ electrolyte and GDC functional layers, with excellent contact between layers. The porous electrode layer with 20 µm in thickness, facilicates gas transfer. The fuel electrode underwent full reduction in 3%H_2_O─H_2_ for 2 h. Open circuit voltages from 700 °C to 800 °C were around 1 V. The peak power densities for the acid‐etched cell are 1.31, 1.00, and 0.68 W cm^−2^ at 800 °C, 750 °C, and 700 °C, respectively. The maximum peak power density (1.31 W cm^−2^) is 1.47 times higher than that of the unetched cell (0.89 W cm^−2^). To further elucidate these improvements, the distribution of relaxation time (DRT) was conducted as depicted in Figure S9 (Supporting Information).^[^ ^33^ ^]^ The low frequency (LF) peak indicates gas diffusion and conversion, while the high frequency (HF) peak represents O^2−^ conduction in the electrolyte and GDC|SSZ|electrodes interfaces. Middle frequency (MF) peaks denote gas ad/desorption and charge transfer in the electrodes.^[^ ^34^ ^]^ The MF peaks dominate the whole electrochemical process for both cells, underscoring that charge transfer and H_2_ oxidation reactions are the rate‐limiting steps.^[^ ^35^ ^]^ Notably, the most significant decreased MF peaks is attributed to the larger amount of CoFe NPs, which are expected to optimize the H_2_ adsorption, activation, and dissociation processes.^[^ ^36^ ^]^ The fuel cell performance with the SCFM‐A‐GDC fuel electrode is comparable to the recent fuel cells with other highly active perovskite fuel electrodes (Table S7, Supporting Information).^[^ ^9^, ^11^, ^16^, ^19^, ^26^, ^36^, ^37^ ^]^

### 2.4 CO_2_ Electrolysis Cell Mode Performance

**Figure 4:**
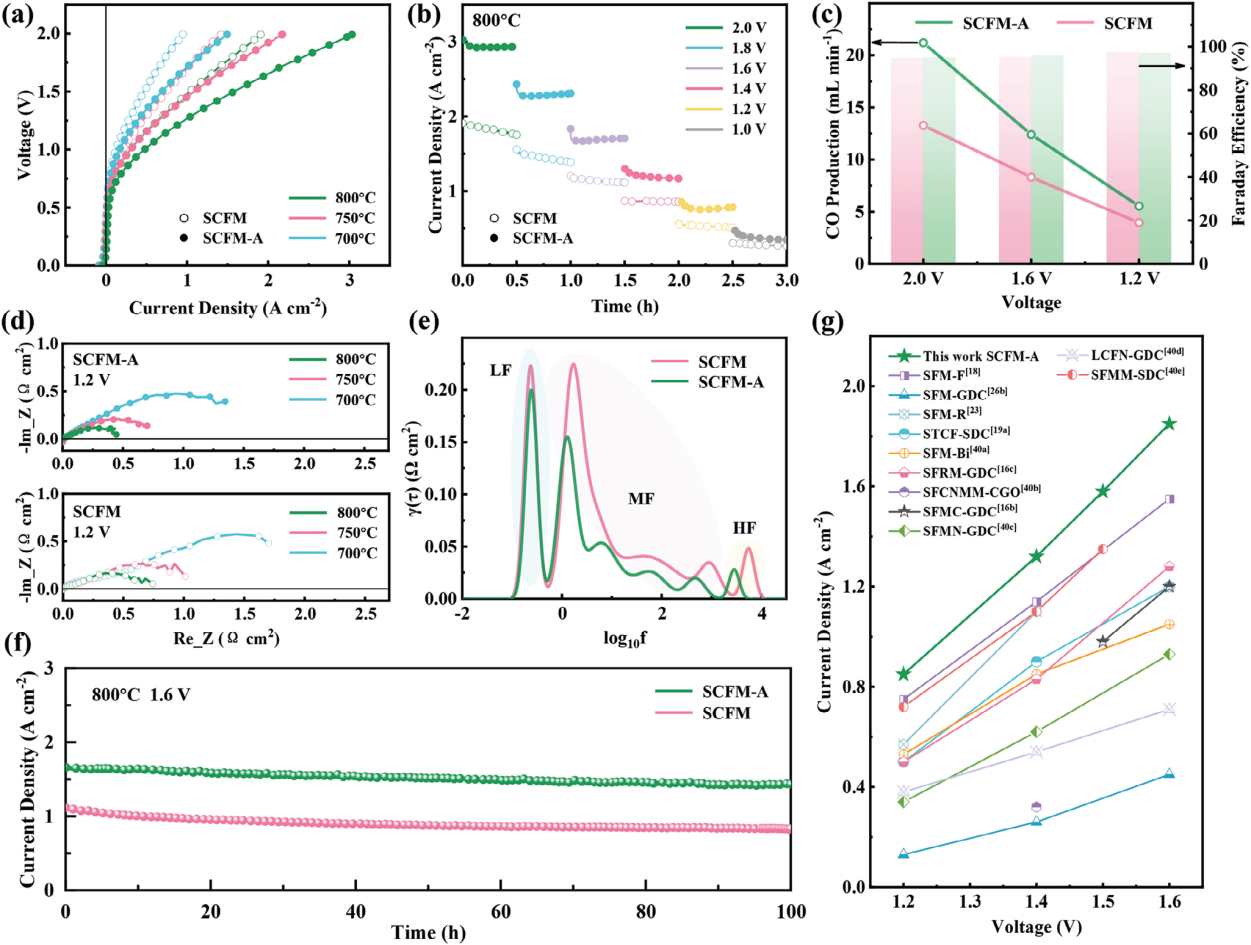
Single cell EC mode performance: a) *I–V* curves with SCFM and SCFM‐A as fuel electrode. b) Short time stability under different voltages. c) Faraday efficiency and CO production under different voltages. d) Nyquist plots for EIS under working voltage of 1.6 V. e) Corresponding DRT results. f) 100 h stability test under 1.6 V at 800 °C. g) Comparison in current density obtained under various voltages at 800 °C for different fuel electrodes and electrolytes.

The single cells were then tested for CO_2_ electrolysis, with the fuel electrode (cathode) fed pure CO_2_ and the oxygen electrode exposed to air. Tests were conducted from 700 to 800 °C, followed by short‐time stability step test and long‐time stability test at 800 °C at different voltages. As shown in **Figure** **4**, the acid‐etched cell achieved 3.03 A cm^−2^ under 2.0 V and 1.85 A cm^−2^ under 1.6 V at 800 °C, making a 56% increase compared to the unetched cell. The production was identified via gas chromatography, and Faraday efficiency for CO exceeded 95% across different operating voltages. Polarization resistance and DRT results are presented in Figure 4d,e and Figure S10 (Supporting Information), showing declines mainly in HF and MF peaks. The HF peak primarily reflects O^2−^ conduction in the electrodes and interfaces, while MF peaks signify the CO_2_RR and electron transfer in the electrodes. Acid etching increases the A/B ratio and oxygen vacancies, boosting CoFe NPs exsolution and O^2−^ conduction in the electrodes.^[^ ^8a^ ^]^ This improvement also enhances CO_2_ adsorption, CO_2_RR efficiency, and overall electrolysis cell performance. Stability test over 100 h reveals both cells remain stable, with minor degradation due to the RP perovskite structure after reduction.^[^ ^38^ ^]^ The unetched cell exhibits stronger degradation in the first 20 h, possibly attributed to the more unfavorable surface Sr (SrO and SrCO_3_), which can be etched away by acid.^[^ ^39^ ^]^ The reduced SCFM‐A‐GDC fuel electrode has exhibited its potential of the efficient conversion for CO_2_, indicated by its comparable current density among the other perovskite‐based electrolysis cells reported recently (Table S8, Supporting Information).^[^ ^16^, ^18^, ^19^, ^23^, ^26^, ^40^ ^]^

The weight loss in O_2_ from 200 °C to 800 °C was measured and illustrated in Figure S11 (Supporting Information). SCFM‐A exhibits a significantly higher weight loss of 3.33% compared to 1.77% for SCFM, indicating that more oxygen vacancies are formed.^[^ ^41^ ^]^ H_2_‐temperature programmed reduction (H_2_‐TPR) was conducted to probe oxide reducibility of SCFM and SCFM‐A, as shown in Figure S12 (Supporting Information). In the temperature range of 100–600 °C, the reduction peaks correspond to the transition from Co^3+^ to Co^2+^, Fe^3+^ to Fe^2+^, and Mo^6+^ to Mo^5+^. At higher temperature of 600–800 °C, Co^2+^ is more readily reduced to its 0‐valence state compared to Fe^2+^.^[^ ^36^, ^42^ ^]^ The higher intensity of the hydrogen absorption peak above 800 °C corresponds to increased exsolved CoFe NPs. These results prove that acid etching induced A‐deficient surface facilitates the exsolution of CoFe alloy NPs. The adsorption and activation of CO_2_ on the fuel electrode surface are usually quite poor for the lack of polarity of linear molecules.^[^ ^40c^ ^]^ CO_2_‐temperature programmed desorption (CO_2_‐TPD) results in Figure S13 (Supporting Information) reveals different adsorption peaks between the two samples. Peak below 200 °C indicates physically adsorbed CO_2_, while peak around ≈400 °C is attributed to decomposition products of chemically adsorbed CO_2_, including monodentate, bidentate, tridentate and carbonates.^[^ ^43^ ^]^ SCFM‐A exhibited an additional peak around ≈730 °C, suggesting enhanced bonding of adsorbed CO_2_.^[^ ^44^ ^]^ The presence of more CoFe alloy NPs in the etched sample creates new oxygen vacancies and active adsorption sites, enhancing CO_2_ adsorption and thereby improving CO_2_RR performance in SOCs.

## 3 Conclusion

We develop a nanosurface reconstruction strategy to engineer an active hierarchical structured fuel electrode with excellent SOCs electrochemical performance under both FC and EC modes. Acid etching effectively reconstructs the perovskite surface, leading to the A‐site deficiency, B‐site exposure and rich oxygen vacancies. Such a reconstruction strategy surprisingly promotes the ion conduction, diffusion and exsolution, thereby accelerating kinetics of both hydrogen oxidation reaction and CO_2_ reduction reaction. The etched fuel electrode SCFM‐A exhibits more CoFe NPs post‐reduction, resulting in enhanced electrochemical catalytic performance in SOCs. These findings not only provide a simple approach to rationally fabricate the nanosurface‐reconstructed catalyst by selective etching, but also significantly boost the electrochemical performance in SOCs.

## 4 Experimental Section

### Sample Preparation

Sr_2_Co_0.4_Fe_1.2_Mo_0.4_O_6‐δ_ (SCFM) particles were synthesized by the sol‐gel method using stoichiometric Sr(NO_3_)_2_, Co(NO_3_)_2_ 6H_2_O, Fe(NO_3_)_3_ 9H_2_O and (NH_4_)_6_Mo_7_O_24_ 4H_2_O. The obtained xerogel was calcined at 1000 °C for 5 h in air to acquire the SCFM pristine particles. La_0.6_Sr_0.4_Co_0.2_Fe_0.8_O_3‐δ_ (LSCF) particles were prepared via the method as well.

### Cell Fabrication

First, Gd_0.1_Ce_0.9_O_1.95_ (GDC), ethyl cellulose, and terpineol were mixed and fully ball‐milled to prepare a well‐dispersed suspension. The GDC suspension was then spin‐coated on the both side of scandia‐stabilized zirconia electrolyte (SSZ, ≈250 µm thickness) and calcined to form a ≈3 µm GDC barrier layer. The second procedure was screen‐printing the SCFM‐GDC and LSCF‐GDC inks on the GDC interlayer separately, and then calcined at 1000 °C for 3 h, fabricating the five‐layers sandwich‐structured SCFM‐GDC|GDC|SSZ|GDC|LSCF‐GDC single cells. At last, both electrodes were covered with silver paste and silver wires as the current collection.

### Acid Etching Procedure

Acid etching procedure is illustrated in Figure S14 (Supporting Information). The glacial acetic acid (99.5%, Aladdin) was dropped into the SCFM fuel electrode on the heater and then washed the etched electrode by deionized water to get the acid etched SCFM. The selection of etching temperature and time was determined by the structural and morphological results from Figure S1 (Supporting Information). The X‐ray diffraction (XRD) results show that the lattice structure stays the same as unetched after the three different treatment procedures. 100 °C‐1 h was chosen and denoted as SCFM‐A for the proper etched morphology, compared with the over‐etching of 200 °C‐1 h and no obvious mark of 100 °C‐0.5 h.

### Sample Characterization

The phase structure was confirmed by powder XRD (D8 ADVANCEA25) at room temperature. XRD data was analyzed and refined using FullProf software. The Raman spectra were collected by a Renishaw in‐Via Raman microscope. Morphology of powders and single cell were conducted by scanning electron microscopy (SEM, Gemini SEM 500) and transmission electron microscopy (TEM, FEI Talos F200X).

The inductively coupled plasma atomic emission spectroscopy (ICP‐OES) results of the samples were analyzed by Agilent 5110. The X‐ray photoelectron spectroscopy (XPS) was conducted on Thermo Fisher ESCALAB Xi+. The X‐ray absorption spectroscopy (XAS) results of Sr, Co, and Fe K‐edge were collected at BL14W1 beamline of Shanghai Synchrotron Radiation Facility (SSRF). Thermogravimetric Analysis (TGA, STA449C) was adopted at the temperature range of 20–800 °C in pure oxygen. H_2_‐temperature‐programmed reduction (H_2_‐TPR) and CO_2_‐temperature programmed desorption (CO_2_‐TPD) experiments were carried out using a Micromeritics Auto Chem II 2920.

### Electrochemical Measurements

The electrochemical performances were measured by Solarton SI 1287 and SI 1260. The frequency range of electrochemical impedance spectroscopy (EIS) was from 10^−1^ to 10^6^ Hz with a signal amplitude of 10 mV. The distribution of relaxation time (DRT) was conducted via the DRTtools. The power density tests of the single cells were completed with a 30 mL min^−1^ flowrate of wet H_2_. The CO_2_ electrolysis was tested with the cathode fed with pure CO_2_ (30 mL min^−1^) while the anode exposed in the air. The Faradaic efficiency of CO was evaluated by outlet gases from fuel electrode determined by gas chromatography (Agilent, 8890 GC System).

## Conflict of Interest

The authors declare no conflict of interest.

## Author Contributions

Y.S. and J.Z. contributed equally to this work. J.Z. conceived the idea. Y.S. prepared samples, fabricated devices, conducted most of the characterizations and writing the original draft. J.Z., D.N., and K.W. supervised the project and process. J.Y. and J.C. contributed to device performance optimization. Z.L. and Z.Z. assisted in the preparation of electrochemical tests. C.Y. and Z.X. carried out some of the characterizations. All authors discussed the results and contributed to the revisions of the manuscript.

## Supporting information



Supporting Information

## Data Availability

The data that support the findings of this study are available from the corresponding author upon reasonable request.
