# Supplementary material for: Nanosurface‐Reconstructed Fuel Electrode by Selective Etching for Highly Efficient and Stable Solid Oxide Cells
Source: Adv Sci (Weinh). 2024 Dec 3;12(4):2409272. doi: 10.1002/advs.202409272 (PMC11775559; doi:10.1002/advs.202409272)
Supplement: Supplementary file 1 — Supporting Information [file ADVS-12-2409272-s001.pdf]

ADVANCED SCIENCE
Open Access

Supporting Information

for *Adv. Sci.*, DOI 10.1002/advs.202409272

Nanosurface-Reconstructed Fuel Electrode by Selective Etching for Highly Efficient and Stable Solid Oxide Cells

Yueyue Sun, Jun Zhou, Jiaming Yang, Dragos Neagu, Zhengrong Liu, Chaofan Yin, Zixuan Xue, Zilin Zhou, Jiajia Cui* and Kai Wu**

Supporting Information

Nanosurface-reconstructed fuel electrode by selective etching for highly efficient and stable solid oxide cells

Yueyue Sun[1], Jun Zhou[1,], Jiaming Yang, Dragos Neagu, Zhengrong Liu, Chaofan Yin, Zixuan Xue, Zilin Zhou, Jiajia Cui*, Kai Wu**

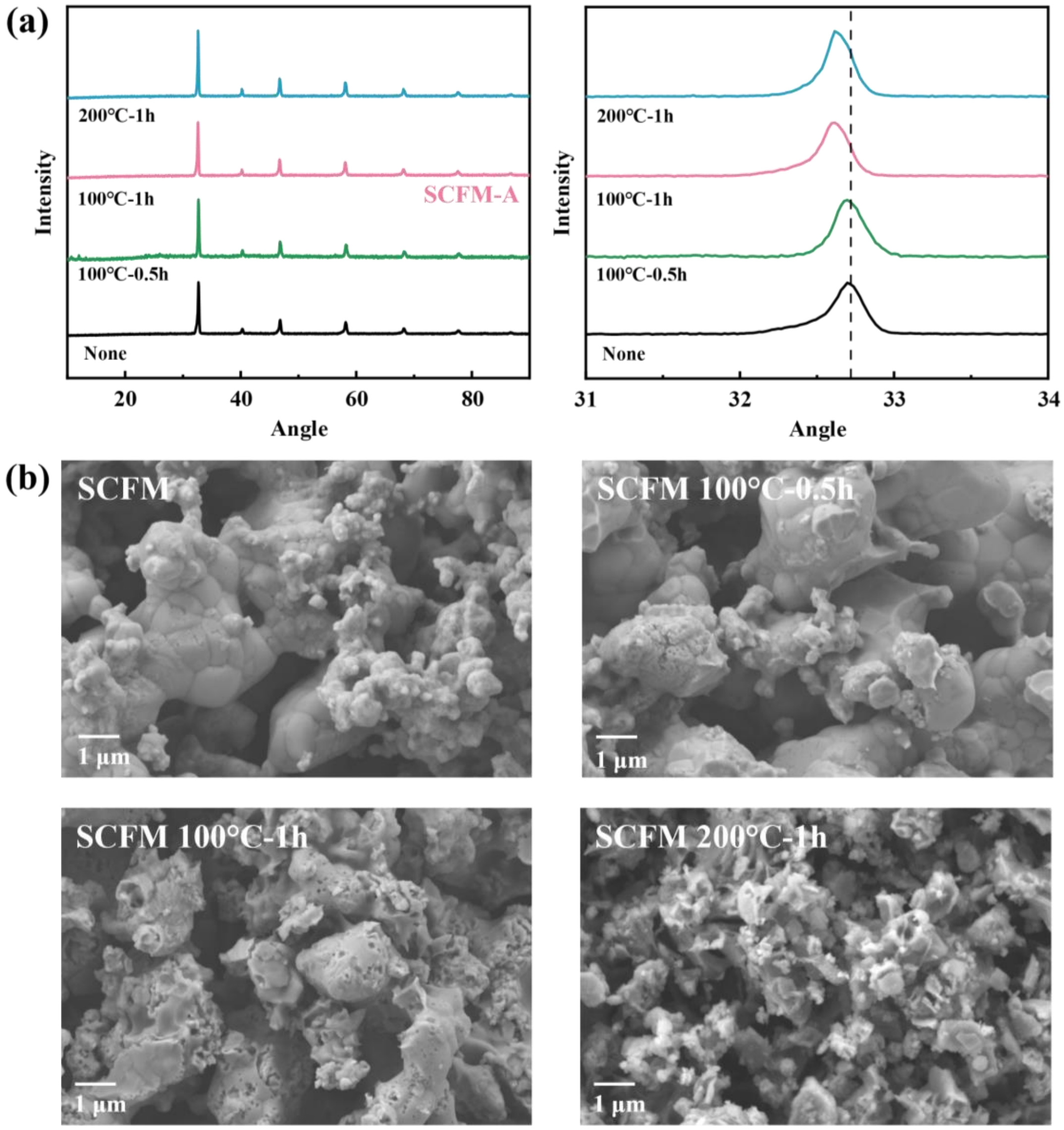

Figure S1. XRD patterns and SEM images of different acid etching temperature and time.

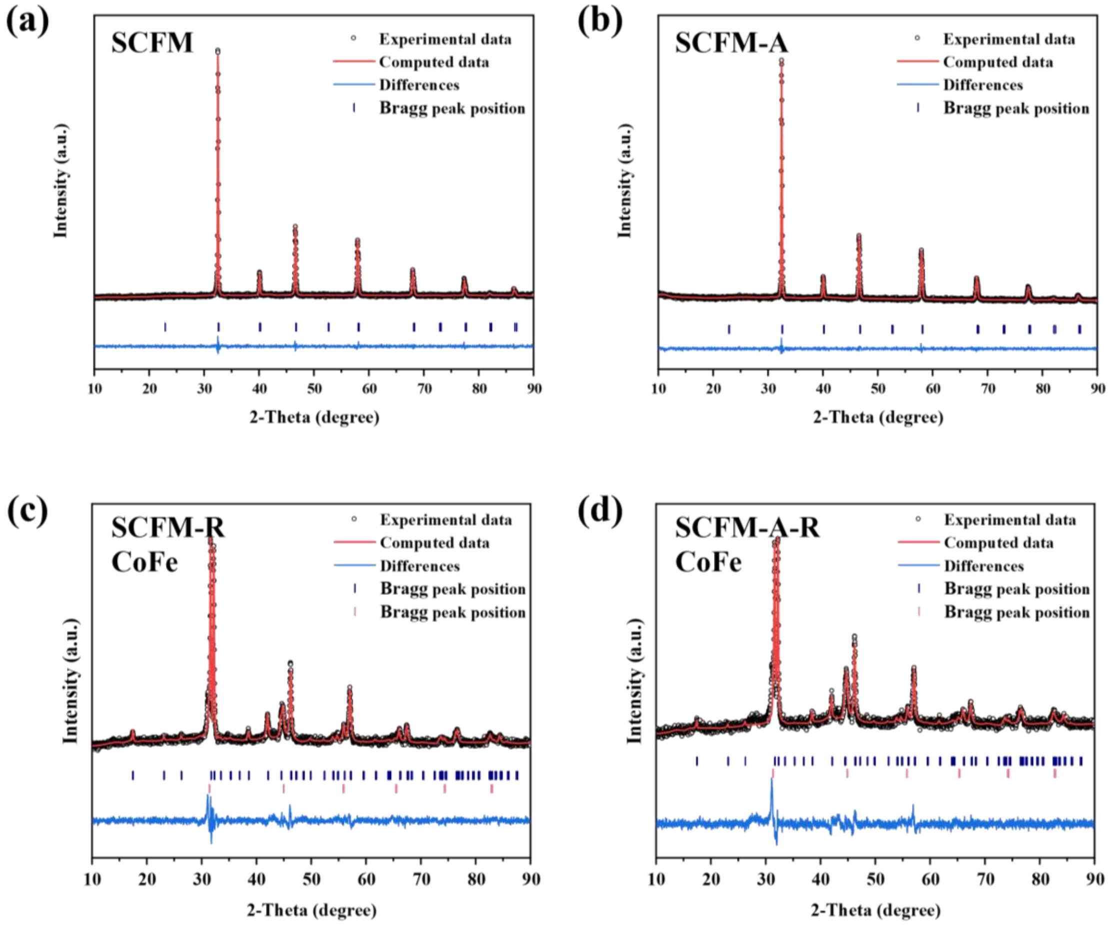

Figure S2. Rietveld refinement profiles of four samples.

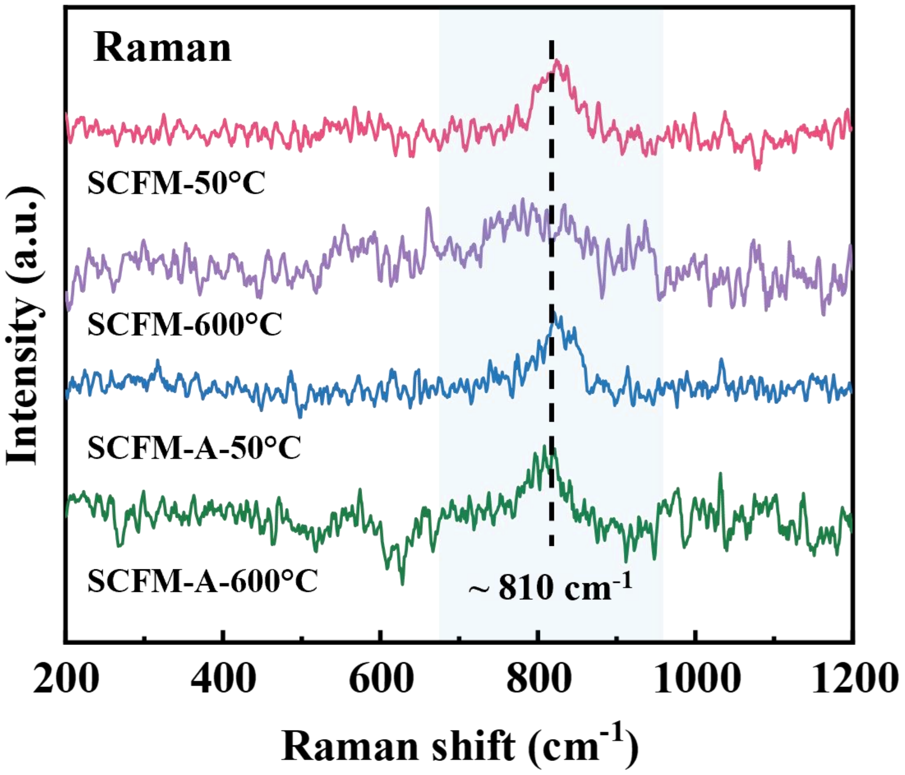

Figure S3. In-situ Raman results of SCFM and SCFM-A at 50°C and 600°C.

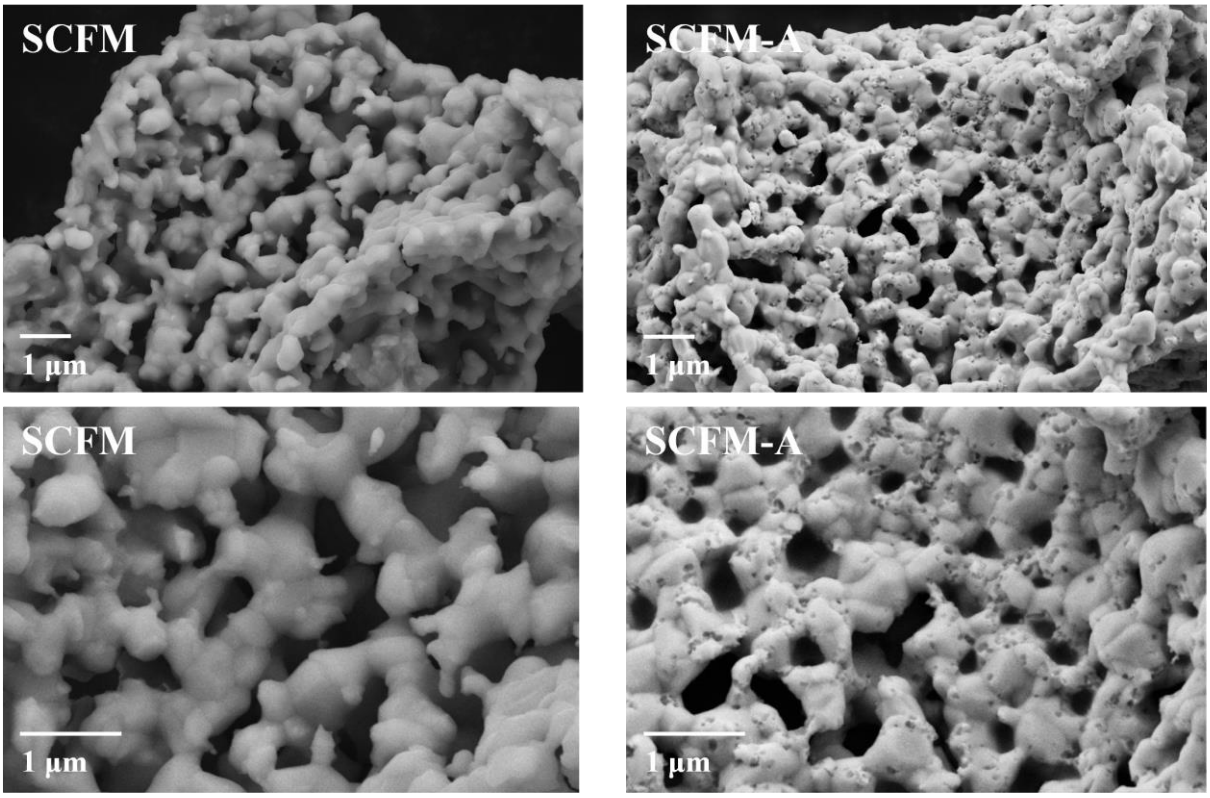

Figure S4. Additional SEM images of SCFM and SCFM-A.

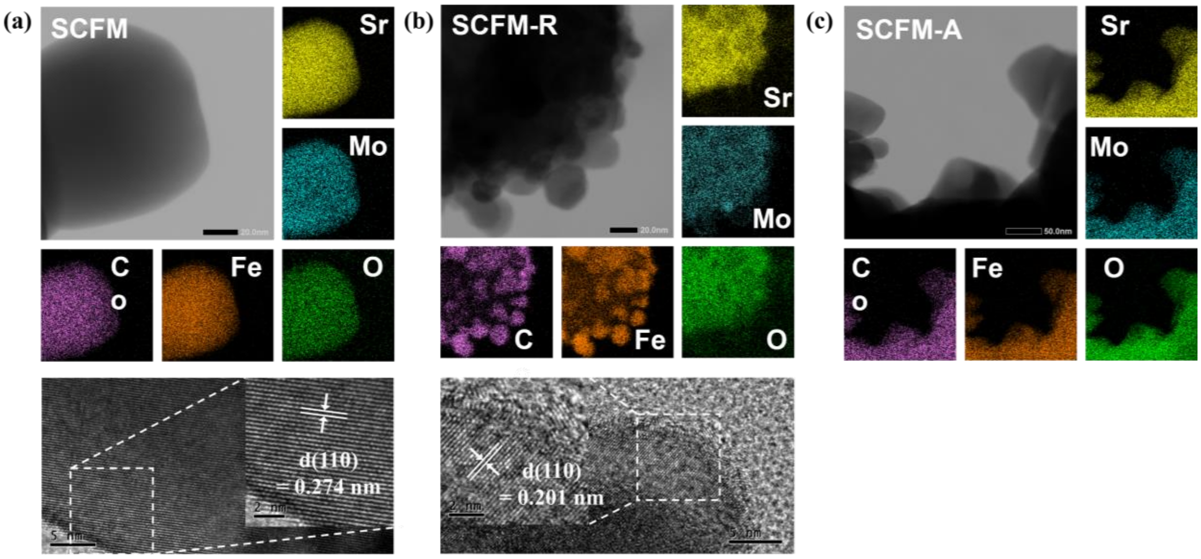

Figure S5. TEM images of SCFM, SCFM-R, and SCFM-A.

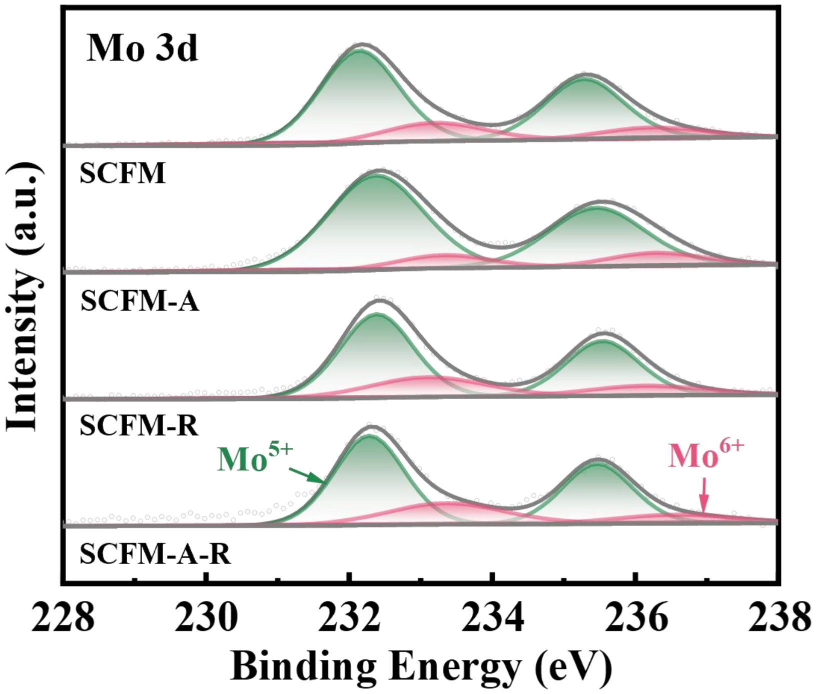

Figure S6. XPS results of Mo 3d.

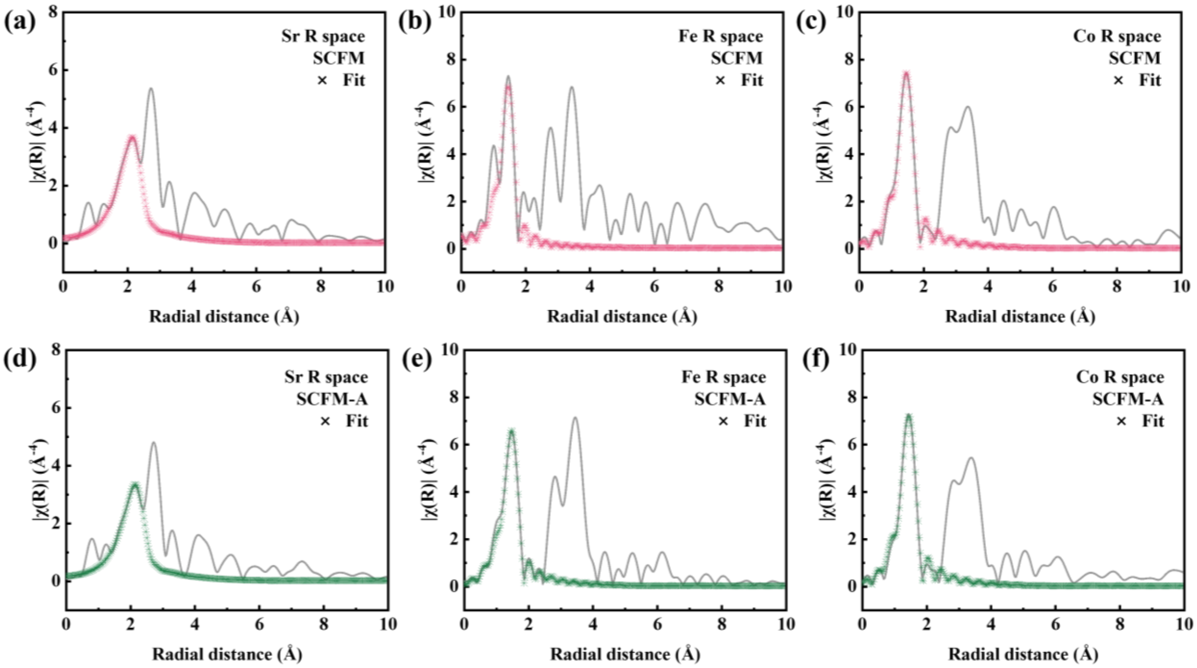

Figure S7. EXAFS fitting curves of Sr, Co, Fe K-edge at R space.

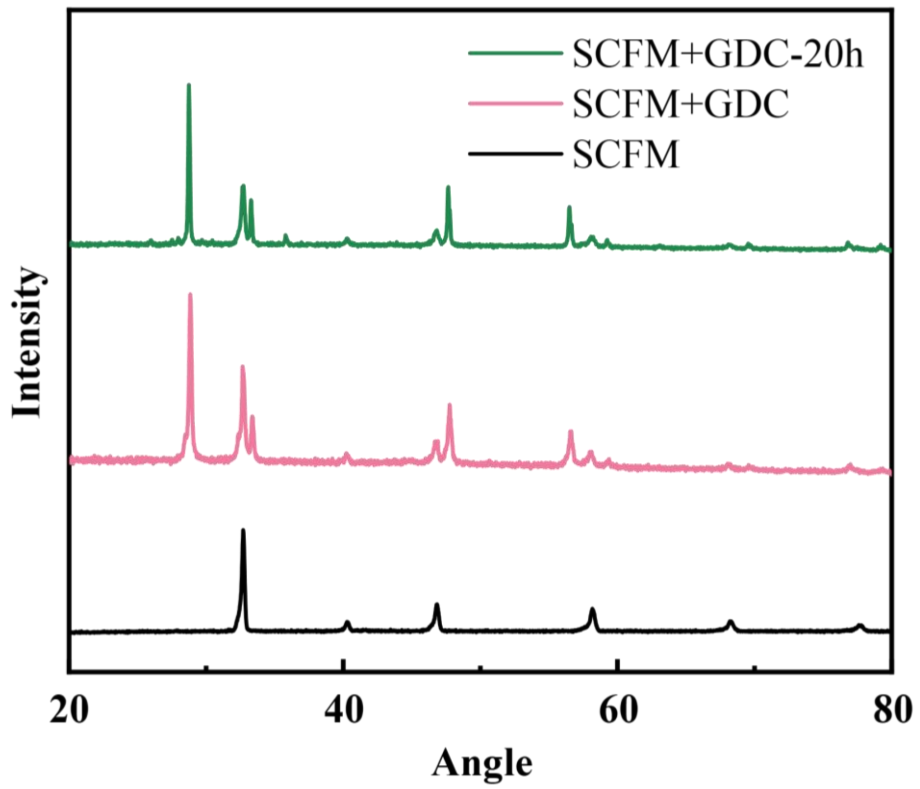

Figure S8. XRD patterns of the composite electrode before and after a 20 h calcination.

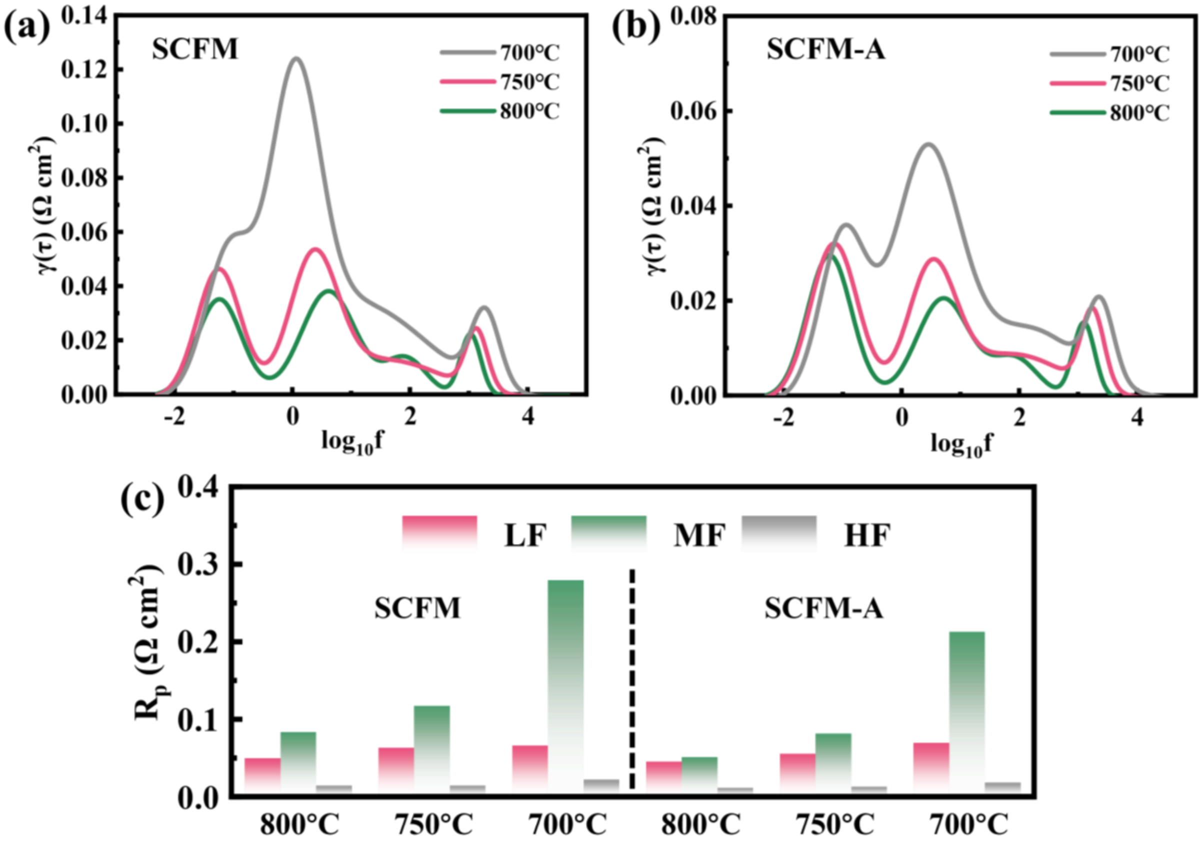

Figure S9. DRT results under FC mode.

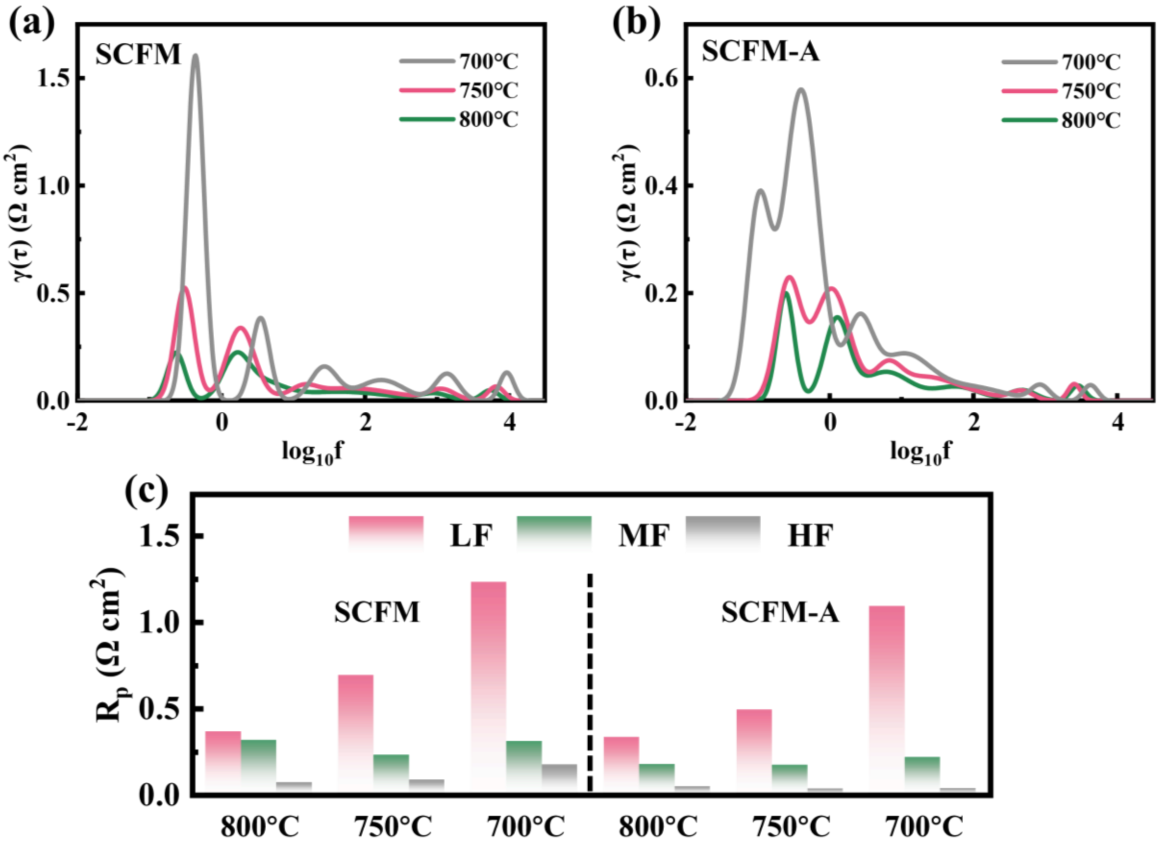

Figure S10. DRT results under EC mode.

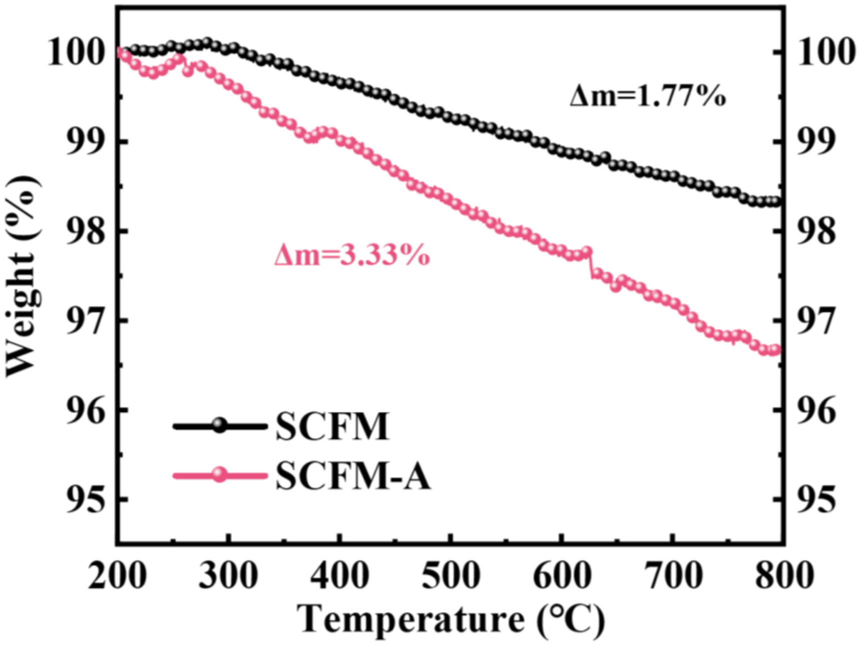

Figure S11. TGA curves of SCFM and SCFM-A.

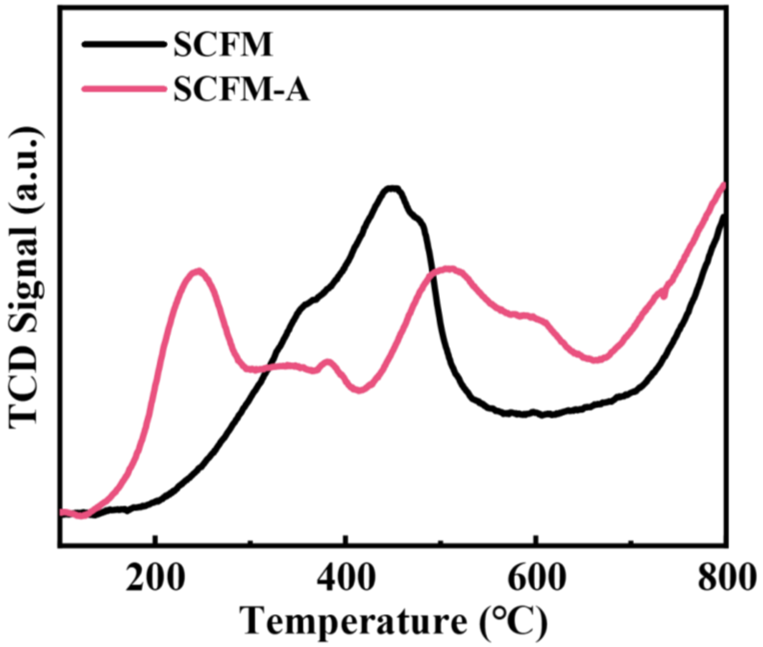

Figure S12. H_2-TPR curves of SCFM and SCFM-A.

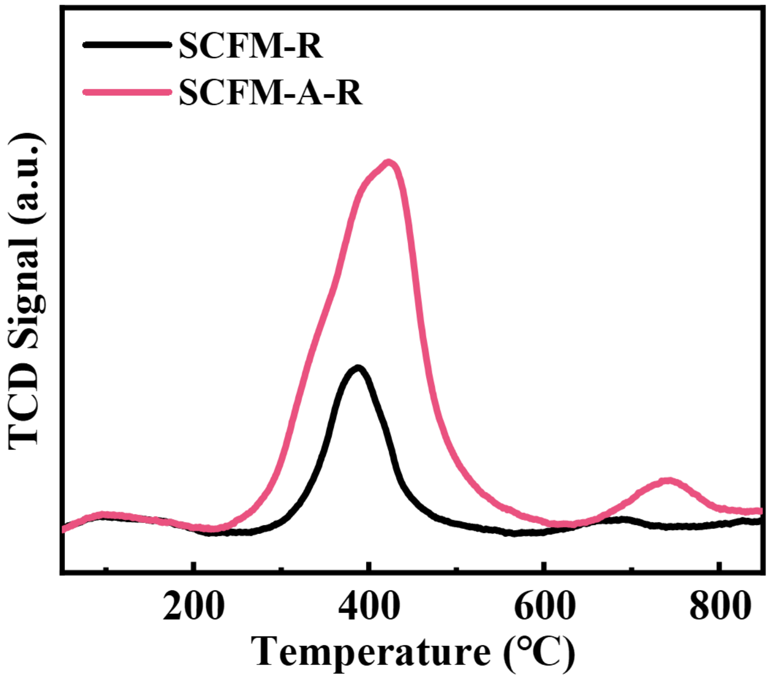

Figure S13. CO_2-TPD curves of SCFM-R and SCFM-A-R.

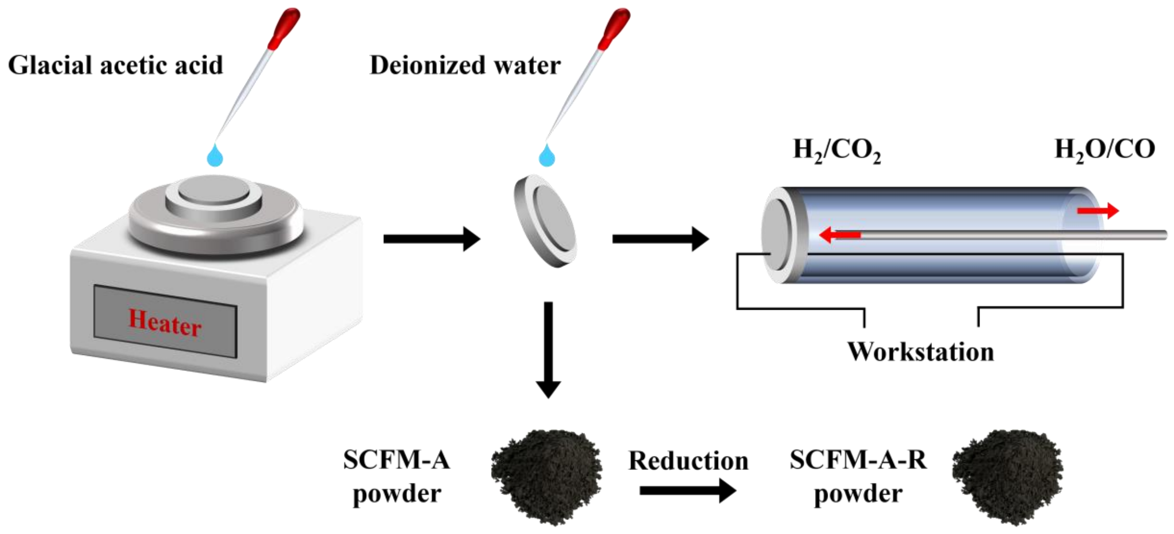

Figure S14. Acid etching procedure.

Table S1. Summary of structural parameters for four samples from Rietveld refinement.

Sample		SCFM	SCFM-A	SCFM-R		SCFM-A-R	
Phase		$Sr_2Co_{0.4}Fe_{1.2}Mo_{0.4}O_{6-\delta}$	$Sr_2Co_{0.4}Fe_{1.2}Mo_{0.4}O_{6-\delta}$	$Sr_3Co_{0.1}Fe_{1.3}Mo_{0.6}O_{7-\delta}$	CoFe	$Sr_3Co_{0.1}Fe_{1.3}Mo_{0.6}O_{7-\delta}$	CoFe
Space group		Pm-3m	Pm-3m	I4/mmm	Pm-3m	I4/mmm	Pm-3m
Lattice parameters [Å]	a	3.8962	3.8963	3.9285	2.8585	3.9285	2.8631
	b	3.8962	3.8963	3.9285	2.8585	3.9285	2.8631
	c	3.8962	3.8963	20.3975	2.8585	20.4134	2.8631
Content [wt%]		100.00	100.00	86.93	13.07	76.28	23.72
Reliability factors	R_p [%]	3.29	2.70	3.23		3.52	
	R_{wp} [%]	4.13	3.40	4.43		5.02	
	R_{exp} [%]	3.79	3.19	3.06		3.22	
	χ^2	1.19	1.14	2.10		2.42	

Table S2. ICP-OES results of SCFM and SCFM-A powders.

Sample	Sr [mol%]	Co [mol%]	Fe [mol%]	Mo [mol%]	A/B
SCFM	49.65	10.06	29.66	10.63	0.99
SCFM-A	49.26	9.82	30.36	10.57	0.97

Table S3. ICP-OES results of washed water for SCFM and SCFM-A.

Sample	Sr [mol%]	Co [mol%]	Fe [mol%]	Mo [mol%]	A/B
SCFM washed water	63.43	1.53	19.47	15.57	1.73
SCFM-A washed water	87.00	10.82	0.58	1.61	6.69

Table S4. XPS elements content results of SCFM and SCFM-A.

Sample	Sr [mol%]	Co [mol%]	Fe [mol%]	Mo [mol%]	A/B
SCFM	64.90	8.63	19.33	7.14	1.85
SCFM-A	55.52	5.29	23.57	15.62	1.25

Table S5. XPS valence states content results of different elements in four samples.

Sample	O [%]			Sr [%]		Co [%]			Fe [%]			Mo [%]	
	lattice	absorbed	molecules	lattice	surface	2+	3+	0	2+	3+	0	5+	6+
SCFM	6	59	35	15	85	31	69	\	49	51	\	81	19
SCFM-A	11	84	5	11	89	25	75	\	52	48	\	86	14
SCFM-R	9	78	13	4	96	42	48	10	32	53	15	75	25
SCFM-A-R	10	80	10	6	94	40	46	14	31	46	23	75	25

Table S6. EXAFS fitting parameters at the Sr, Co, Fe K-edge for SCFM and SCFM-A.

Sample	Shell	$CN^{a)}$	R [Å][b)]	σ^2 [Å^2][c)]	ΔE_0 [eV][d)]	R factor
SCFM	Sr-O	11.98	2.74	0.001±0.003	-0.456±0.463	0.007
	Co-O	5.15	1.92	0.006±0.001	-2.420±0.280	0.001
	Fe-O	5.03	1.93	0.006±0.003	-3.611±0.955	0.009
SCFM-A	Sr-O	11.16	2.74	0.009±0.003	-0.294±0.438	0.008
	Co-O	4.99	1.91	0.006±0.001	-2.827±0.398	0.002
	Fe-O	4.84	1.93	0.006±0.001	-4.953±0.418	0.003

[a)]*CN*, the coordination numbers; [b)]*R*, the bond distance; [c)]σ^2, the Mean Square Relative Displacement (MSRD); [d)]E_0, the inner potential correction; *R* factor indicates the goodness of the fit. Fitting range: $3.0 \leq k$ (/Å) ≤ 12.0, $1.2 \leq R$ (Å) ≤ 2.4, and $S_0^2 = 0.800$. A reasonable range of EXAFS fitting parameters: $0.700 < S_0^2 < 1.000$; $CN > 0$; $\sigma^2 > 0$ Å^2; $|\Delta E_0| < 10$ eV; R factor < 0.02.

Table S7. Comparison of the perovskite fuel electrode SOFCs in this study and those reported in literatures at 800°C.

Fuel electrode	Electrolyte-thickness [μm]	Air electrode	Max power density [W cm^{-2}]	Ref.
$PrBaMn_2O_{5+\delta}$-Pr_6O_{11}	8YSZ	$PrBaMn_2O_{5+\delta}$-Pr_6O_{11}	0.42	[1]
$(Ba_{0.9}La_{0.1})_{0.95}Co_{0.7}Fe_{0.2}Nb_{0.1}O_{3-\delta}$	$Sm_{0.2}Ce_{0.8}O_{2-\delta}$ (SDC)-300	$Ba_{0.5}Sr_{0.5}Co_{0.8}Fe_{0.2}O_{3-\delta}$ (BSCF)-SDC	0.51	[2]
$La_{0.8}Ce_{0.1}Ni_{0.4}Ti_{0.6}O_{3-\delta}$-$CeO_2$	$Zr_{0.89}Sc_{0.1}Ce_{0.01}O_{2-\delta}$ (SSZ)-120	$(La_{0.8}Sr_{0.2})_{0.95}MnO_{3-\delta}$-SSZ	0.64	[3]
$La_{0.43}Sr_{0.37}Fe_{0.09}Cu_{0.03}Ti_{0.88}O_{3-\delta}$	SSZ-85	$(La_{0.8}Sr_{0.2})_{0.95}MnO_{3-\delta}$-SSZ	0.68	[4]
$La_{0.4}Sr_{0.4}Ti_{0.9}Ni_{0.1}O_3$	SSZ-250	$(La_{0.6}Sr_{0.4})_{0.95}Co_{0.2}Fe_{0.8}O_{3-\delta}$ (LSCF)-GDC	0.85	[5]
$Sr_2Ti_{0.8}Co_{0.2}FeO_{6-\delta}$-SDC	$La_{0.8}Sr_{0.2}Ga_{0.83}Mg_{0.17}O_{3-\delta}$ (LSGM)-230	$La_{0.7}Sr_{0.3}CoO_{3-\delta}$-SDC-$PrO_x$	0.58	[6]
$Sr_2FeMo_{0.65}Ni_{0.35}O_{6-\delta}$	LSGM-300	$La_{0.58}Sr_{0.4}Co_{0.2}Fe_{0.8}O_{3-\delta}$	0.79	[7]
$Sr_2Fe_{1.5}Mo_{0.4}Sb_{0.1}O_{6-\delta}$	LSGM-280	LSCF-SDC	0.92	[8]
Ru@Ru-$Sr_2Fe_{1.5}$Mo0.5$O_{6-\delta}$/Ru-GDC	LSGM-230	$PrBa_{0.5}Sr_{0.5}Co_{1.5}$Fe0.5$O_{5+\delta}$-GDC	1.03	[9]
$Sr_2Co_{0.4}Fe_{1.2}Mo_{0.4}O_{6-\delta}$ (SCFM)	SSZ-250	$La_{0.6}Sr_{0.4}Co_{0.2}Fe_{0.8}O_{3-\delta}$-GDC	0.89	This work
Acid etched SCFM	SSZ-250	$La_{0.6}Sr_{0.4}Co_{0.2}Fe_{0.8}O_{3-\delta}$-GDC	1.31	This work

Table S8. Comparison of the current density for CO_2 electrolysis obtained at 800°C under 1.4/1.6 V with various fuel electrodes.

Fuel electrode	Electrolyte-thickness [μm]	Air electrode	Feeding gas	Voltage [V]	Current density [A cm^{-2}]	Ref.
$Sr_2Fe_{1.5}Mo_{0.5}O_{6-\delta}F_{0.1}$	LSGM-250	LSCF-SDC	100% CO_2	1.6	1.55	[10]
$Sr_2Fe_{1.5}Mo_{0.5}O_{6-\delta}$-GDC	8YSZ-500	$(La_{0.75}Sr_{0.25})_{0.95}MnO_{3-\delta}$-YSZ	95% CO_2 + 5% N_2	1.6	0.45	[11]
$Sr_2Fe_{1.5}Mo_{0.5}O_{6-\delta}$	LSGM-230	LSCF-SDC	100% CO_2	1.4	1.10	[12]
$Sr_2Ti_{0.8}Co_{0.2}FeO_{6-\delta}$-SDC	LSGM-230	$La_{0.7}Sr_{0.3}CoO_{3-\delta}$-SDC-$PrO_x$	100% CO_2	1.6	1.20	[6]
$Bi_{0.1}Sr_{1.9}Fe_{1.5}Mo_{0.5}O_{6-\delta}$	LSGM-300	BSCF	100% CO_2	1.6	1.05	[13]
$Sr_2Fe_{1.4}Ru_{0.1}Mo_{0.5}O_{6-\delta}$-GDC	LSGM-500	BSCF-GDC	95% CO_2 + 5% N_2	1.6	1.28	[14]
$Sr_2FeCo_{0.2}Ni_{0.2}Mn_{0.1}Mo_{0.5}O_{6-\delta}Ce_{0.8}Gd_{0.2}O_{2-\delta}$	SSZ-150	LSCF	15% CO_2 + 85% Ar	1.4	0.32	[15]
$Sr_2Fe_{1.35}Mo_{0.45}Co_{0.2}O_{6-\delta}$-GDC	LSGM-600	BSCF-GDC	95% CO_2 + 5% N_2	1.6	1.20	[16]
$Sr_2Fe_{1.35}Mo_{0.45}Ni_{0.2}O_{6-\delta}$-GDC	LSGM-500	LSCF-SDC	95% CO_2 + 5% N_2	1.6	0.93	[17]
$La_{0.6}Ca_{0.4}Fe_{0.8}Ni_{0.2}O_{3-\delta}$-GDC	YSZ-300	$La_{0.6}Ca_{0.4}Fe_{0.8}Ni_{0.2}O_{3-\delta}$-GDC	100% CO_2	1.6	0.71	[18]
$Sr_2Fe_{1.4}Mn_{0.1}Mo_{0.5}O_{6-\delta}$-GDC	LSGM-400	LSCF-SDC	100% CO_2	1.4	1.10	[17]
Acid etched SCFM	SSZ-250	$La_{0.6}Sr_{0.4}Co_{0.2}Fe_{0.8}O_{3-\delta}$-GDC	100% CO_2	1.6	1.85	This work
Acid etched SCFM	SSZ-250	$La_{0.6}Sr_{0.4}Co_{0.2}Fe_{0.8}O_{3-\delta}$-GDC	100% CO_2	1.4	1.32	This work

References

[1] Y. Gu, Y. Zhang, Y. Zheng, H. Chen, L. Ge, L. Guo, *Appl. Catal., B* **2019**, *257*, 117868.
[2] H. Hu, M. Li, H. Min, X. Zhou, J. Li, X. Wang, Y. Lu, X. Ding, *ACS Catal.* **2021**, *12*, 828.
[3] S. He, M. Li, J. Hui, X. Yue, *Appl. Catal., B* **2021**, *298*, 120588.
[4] S. Jo, Y. Han Kim, H. Jeong, C.-h. Park, B.-R. Won, H. Jeon, K. Taek Lee, J.-h. Myung, *Appl. Energy* **2022**, *323*, 119615.
[5] J. Yang, J. Zhou, Z. Liu, Y. Sun, C. Yin, K. Wang, R. Li, Z. Zhou, K. Wu, *J. Power Sources* **2023**, *580*, 233369.
[6] X. Sun, Y. Ye, M. Zhou, H. Chen, Y. Li, P. Chen, D. Dong, Y. Ling, M. Khan, Y. Chen, *J. Mater. Chem. A* **2022**, *10*, 2327.
[7] Z. Du, H. Zhao, S. Yi, Q. Xia, Y. Gong, Y. Zhang, X. Cheng, Y. Li, L. Gu, K. Swierczek, *ACS Nano* **2016**, *10*, 8660.
[8] S. Zhang, K. Zhu, X. Hu, R. Peng, C. Xia, *J. Mater. Chem. A* **2021**, *9*, 24336.
[9] F. Hu, K. Chen, Y. Ling, Y. Huang, S. Zhao, S. Wang, L. Gui, B. He, L. Zhao, *Adv. Sci.* **2024**, *11*, 2306845.
[10] Y. Li, Y. Li, Y. Wan, Y. Xie, J. Zhu, H. Pan, X. Zheng, C. Xia, *Adv. Energy Mater.* **2019**, *9*, 1803156.
[11] H. Lv, Y. Zhou, X. Zhang, Y. Song, Q. Liu, G. Wang, X. Bao, *J. Energy Chem.* **2019**, *35*, 71.
[12] X. Xi, J. Liu, W. Luo, Y. Fan, J. Zhang, J. L. Luo, X. Z. Fu, *Adv. Energy Mater.* **2021**, *11*, 2102845.
[13] M. Yang, Z. Yao, S. Liu, J. Wang, A. Sun, H. Xu, G. Yang, R. Ran, W. Zhou, G. Xiao, Z. Shao, *J.Mater. Sci. Technol.* **2023**, *164*, 160.
[14] H. Lv, L. Lin, X. Zhang, R. Li, Y. Song, H. Matsumoto, N. Ta, C. Zeng, Q. Fu, G. Wang, X. Bao, *Nat. Commun.* **2021**, *12*, 5665.
[15] A. López-García, L. Almar, S. Escolástico, A. B. Hungría, A. J. Carrillo, J. M. Serra, *ACS Appl. Energy Mater.* **2022**, *5*, 13269.
[16] H. Lv, L. Lin, X. Zhang, Y. Song, H. Matsumoto, C. Zeng, N. Ta, W. Liu, D. Gao, G. Wang, X. Bao, *Adv. Mater.* **2020**, *32*, 1906193.
[17] H. Lv, L. Lin, X. Zhang, D. Gao, Y. Song, Y. Zhou, Q. Liu, G. Wang, X. Bao, *J. Mater. Chem. A* **2019**, *7*, 11967.
[18] Y. Tian, L. Zhang, Y. Liu, L. Jia, J. Yang, B. Chi, J. Pu, J. Li, *J. Mater. Chem. A* **2019**, *7*, 6395.
